# Spermatogenesis Studies Reveal a Distinct Nonsense-Mediated mRNA Decay (NMD) Mechanism for mRNAs with Long 3′UTRs

**DOI:** 10.1371/journal.pgen.1005979

**Published:** 2016-05-05

**Authors:** Oliver Mühlemann

**Affiliations:** Department of Chemistry and Biochemistry, University of Bern, Bern, Switzerland; University of Basel, SWITZERLAND

Extensive alternative splicing and polyadenylation of pre-mRNAs not only expands the protein coding potential of our genomes but also generates a wealth of mRNA isoforms with different 3′ untranslated regions (UTRs) [1,2]. Since 3′UTRs are major regulators of mRNA stability, localization, and translation, the tissue-specific, developmentally regulated, and stress-induced generation of alternative 3′UTRs greatly contributes to the posttranscriptional regulation of gene expression. It is an intriguing observation that mRNAs with the longest 3′UTRs are predominately present in the brain, whereas the testis is enriched in mRNA isoforms with shorter 3′UTRs [3,4]. In general, mRNAs with shorter 3′UTRs tend to be more stable because (i) they contain fewer binding sites for decay-inducing miRNAs or RNA-binding proteins (RBPs) and (ii) long 3′UTRs can trigger nonsense-mediated mRNA decay (NMD) [1,5].

The term NMD was initially coined to describe the accelerated degradation observed for mRNAs with nonsense mutations that prematurely truncate the open reading frame (ORF) [6]. NMD serves an important cellular quality control function by reducing the production of potentially deleterious C-terminally truncated proteins. However, genome-wide studies uncovered that beyond degrading aberrant mRNAs harboring premature translation termination codons (PTCs), NMD also targets many “normal” mRNAs encoding apparently full-length functional proteins, suggesting a broader biological function in posttranscriptional gene regulation [5,7]. The three conserved core factors (UPF1, UPF2, and UPF3) and additional metazoan-specific proteins are required for NMD in mammalian cells and, although the exact molecular mechanism of NMD is not known, inefficient or aberrant translation termination seems to be a key trigger for NMD. It has been empirically found that exon–exon junctions located >50 nucleotides downstream of the termination codon often trigger NMD, which is typically the case in aberrant PTC-containing transcripts, and that long 3′UTRs can also elicit NMD, a feature found in many of the PTC-free NMD targets [8–10]. Whether these two groups of NMD substrates are recognized and degraded by a common mechanism or whether they employ mechanistically distinct branches of NMD is a major unresolved question in the field.

**Fig 1:**
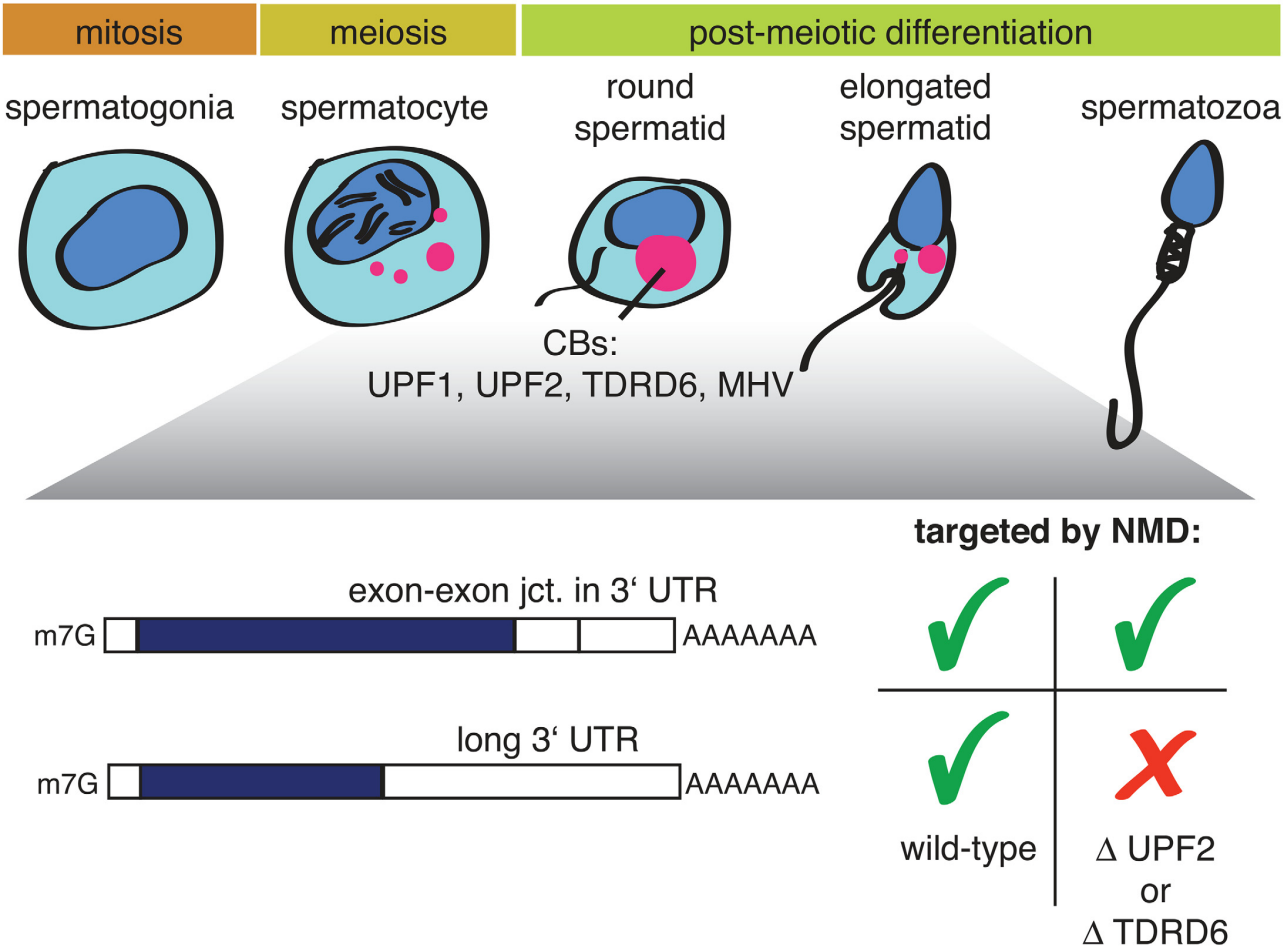
Illustration depicting mammalian spermatogenesis and the effect of a UPF2 or TDRD6 knockout on two different types of NMD-targeted mRNAs. Precursors of chromatid bodies (CBs: red) begin to form in late-stage spermatocytes at the end of meiosis and condense into the typical CB structure in early round spermatids. CBs are enriched in different classes of RNA, NMD factors, and additional proteins, including TDRD6 and MHV.

Two new studies on mouse spermatogenesis [11,12], both published in this issue of *PLOS Genetics*, now shed some unexpected new light on this question. During spermatogenesis, spermatogonia differentiate into spermatocytes, which undergo meiosis and postmeiotically develop into round and, further, elongated spermatids (Fig 1).

Both papers report that the NMD factors UPF1 and UPF2 are highly expressed in postmeiotic spermatocytes and spermatids, where they are found in germ cell-specific perinuclear structures called chromatin bodies (CBs). CBs consist of RNA, many RNA-binding proteins, helicases, and several members of the TUDOR-domain protein family (TDRDs); furthermore, CBs are best known for their role in piRNA biogenesis [13,14]. A conditional UPF2 knockout in spermatogonia led to infertile mice with small testes [11], resembling the “Sertoli-only syndrome” in humans; ablation of TDRD6, which disrupts CB formation and arrests spermatogenesis, gave a similar phenotype. Transcriptome profilings of UPF2 or TDRD6 knockout spermatocytes and round spermatids revealed in both cases a strong enrichment of transcripts with long 3′UTRs among the up-regulated RNAs, while the classical NMD targets (defined by the presence of an exon–exon junction >50 nucleotides downstream of the termination codon) were largely unaffected [11,12]. The UPF2 knockout data is reminiscent of a previous study showing that NMD induced by the presence of exon junction complex (EJC) factors eIF4A3, Y14, and MAGOH downstream of the PTC does not require UPF2 [15]. Together with the results from Bao and colleagues [11], this finding provides evidence for the existence of mechanistically different modes of NMD acting on different types of transcripts and, in particular, strongly suggests a UPF2-independent route of NMD for transcripts with EJCs in the 3′UTR. Because UPF2 is thought to play a crucial role in NMD by promoting the SMG1-mediated phosphorylation of UPF1 [5], it remains, however, unclear how UPF1 phosphorylation could be achieved in the postulated UPF2-independent NMD mode. That the TDRD6 knockout affects the transcriptome in the same way as the UPF2 knockout suggests that there must be a germ-line–specific aspect to NMD. Fanourgakis and colleagues provide several lines of evidence that, in spermatocytes and round spermatids, NMD occurs in the CBs [12]. The absence of TDRD6 disrupted CB formation, UPF1 no longer bound to UPF2 and to the CB component MHV (a Vasa-like helicase), and UPF1 and UPF2 associations with long 3′UTR-containing mRNAs was reduced. Taken together, these observations imply that intact CBs are a prerequisite for this branch of NMD [12]. In contrast, the other NMD branch targeting mRNAs with EJCs in the 3′UTR does not require intact CBs because it was not affected by the TDRD6 knockout. It is noteworthy that piRNA biogenesis was not affected in the TDRD^-/-^cells either.

In summary, the two new studies [11,12] corroborate the view that what is currently called NMD might in fact represent more than one mechanistically distinct mRNA degradation pathway by genetically separating the UPF2- and TDRD6-dependent degradation of mRNAs with long 3′UTRs from the UPF2- and TDRD6-independent degradation of mRNAs with EJCs in the 3′UTR. It will be a future challenge to elucidate the exact mechanisms of the two different NMD routes. The two studies also establish an essential role for NMD in mammalian spermatogenesis by contributing to shaping the male-germ-cell–specific transcriptome, which is typified by mRNAs with unusually short 3′UTRs. While germ-line–specific alternative polyadenylation factors ensure that important mRNAs for sperm development are expressed with a short 3′UTR, a UPF2- and TDRD6-dependent branch of NMD clears ubiquitously expressed mRNAs with long 3′UTRs in these cells.
